# Understanding How Virtual Reality Can Support Mindfulness Practice: Mixed Methods Study

**DOI:** 10.2196/16106

**Published:** 2020-03-18

**Authors:** Elizabeth Seabrook, Ryan Kelly, Fiona Foley, Stephen Theiler, Neil Thomas, Greg Wadley, Maja Nedeljkovic

**Affiliations:** 1 Centre for Mental Health Swinburne University of Technology Hawthorn, Melbourne Australia; 2 School of Computing and Information Systems University of Melbourne Melbourne Australia; 3 Department of Psychological Sciences Swinburne University of Technology Melbourne Australia

**Keywords:** virtual reality, mindfulness, emotion, pilot projects, acceptability, evaluation

## Abstract

**Background:**

Regular mindfulness practice has been demonstrated to be beneficial for mental health, but mindfulness can be challenging to adopt, with environmental and personal distractors often cited as challenges. Virtual reality (VR) may address these challenges by providing an immersive environment for practicing mindfulness and by supporting the user to orient attention to the present moment within a tailored virtual setting. However, there is currently a limited understanding of the ways in which VR can support or hinder mindfulness practice. Such an understanding is required to design effective VR apps while ensuring that VR-supported mindfulness is acceptable to end users.

**Objective:**

This study aimed to explore how VR can support mindfulness practice and to understand user experience issues that may affect the acceptability and efficacy of VR mindfulness for users in the general population.

**Methods:**

A sample of 37 participants from the general population trialed a VR mindfulness app in a controlled laboratory setting. The VR app presented users with an omnidirectional video of a peaceful forest environment with a guided mindfulness voiceover that was delivered by a male narrator. Scores on the State Mindfulness Scale, Simulator Sickness Questionnaire, and single-item measures of positive and negative emotion and arousal were measured pre- and post-VR for all participants. Qualitative feedback was collected through interviews with a subset of 19 participants. The interviews sought to understand the user experience of mindfulness practice in VR.

**Results:**

State mindfulness (*P*<.001; Cohen *d*=1.80) and positive affect (*P*=.006; *r*=.45) significantly increased after using the VR mindfulness app. No notable changes in negative emotion, subjective arousal, or symptoms of simulator sickness were observed across the sample. Participants described the user experience as relaxing, calming, and peaceful. Participants suggested that the use of VR helped them to focus on the present moment by using visual and auditory elements of VR as attentional anchors. The sense of presence in the virtual environment (VE) was identified by participants as being helpful to practicing mindfulness. Interruptions to presence acted as distractors. Some uncomfortable experiences were discussed, primarily in relation to video fidelity and the weight of the VR headset, although these were infrequent and minor.

**Conclusions:**

This study suggests that an appropriately designed VR app can support mindfulness practice by enhancing state mindfulness and inducing positive affect. VR may help address the challenges of practicing mindfulness by creating a sense of presence in a tailored VE; by allowing users to attend to visual and auditory anchors of their choice; and by reducing the scope of the content in users’ mind-wandering. VR has the unique capability to combine guided mindfulness practice with tailored VEs that lend themselves to support individuals to focus attention on the present moment.

## Introduction

### Background

Originating in Buddhist traditions, mindfulness involves an individual focusing his or her attention on the present moment and approaching experiences with a nonjudgmental, nonreactive, and accepting attitude [1,2]. Mindfulness often involves bringing one’s attention to an anchor, most commonly the breath, to facilitate the awareness of a moment-by-moment experience [3].

Mindfulness practice has been demonstrated to be beneficial for mental health by helping people strengthen attention flexibility and adopt an orientation toward experiences that reduces the reliance on automatic thoughts or maladaptive emotion regulation strategies [2,4-6]. However, mindfulness requires conscious effort and can be difficult to maintain, particularly for novice meditators who already expend greater cognitive resources to control their self-regulatory skills [7-9]. Anderson et al [10] recently summarized a range of experiential challenges that can arise during mindfulness practice. These include affective demands such as cognitive effort and frustration, task demands introduced by the physical environment (eg, noisy surroundings and people), and negative emotional or psychological outcomes, such as boredom, upsetting thoughts, and emotions. Adapting mindfulness practice to reduce the likelihood or impact of these challenges may be an important consideration for improving the success of mindfulness-based interventions.

In recent years there has been an interest in using digital technology to support mindfulness practice [11,12]. For example, Web-based interventions have been designed to improve mindfulness skills [13-15], and smartphone apps now deliver guided audio practices that support user engagement with mindfulness through habit formation [11,16,17]. Although these delivery platforms improve the accessibility of mindfulness-based interventions, research has shown that adherence with Web and mobile programs is often low [18], suggesting they may not be engaging over the long term [19]. It is possible that these technologies do not meaningfully assist users with overcoming some of the experiential challenges associated with mindfulness practice, which may contribute to disengagement or even adverse effects [20,21].

Virtual reality (VR) has recently been proposed as a medium to support mindfulness [22,23]. VR technologies may pragmatically address the challenges related to environmental distraction by providing an immersive, engaging, and controlled (ie, predictable) visual and auditory sandbox in which one could rehearse mindfulness skills [12,24,25], shifting attention away from the real-world environment. VR refers to the use of a headset with a display that projects an interactive, audiovisual 360-degree virtual environment (VE) to the user [26-28]. Although VEs can be presented via other mediums (eg, a computer screen), VR systems have greater immersive capacity, which aids in stimulating multiple senses and may create a sense of “presence,” ie, a feeling of *being there* in a simulated environment [27-29]. Presence contributes to an “illusion of reality” in which the user behaves as if the environment were real even though it is computer generated [30,31].

A number of studies have evaluated VR-supported mindfulness practice in relation to its impact on mental health or state mindfulness [12,24,32-34]. In an evaluation with expert meditators from a nonclinical population, Navarro-Haro et al [12] demonstrated that a VR-supported mindfulness practice increased state mindfulness from pretest to posttest, reduced feelings of sadness and anxiety, and increased feelings of relaxation [12]. More recently, Chandrasiri et al [24] evaluated an omnidirectional video recording of a beach scene delivered in VR, which was matched with a breath-focused mindfulness practice in a novice, nonclinical adult sample. Consistent with the findings of Navarro-Haro et al [12], Chandrasiri et al [24] found that state mindfulness significantly increased after using VR. However, their work provides limited insight into how and why the features of VR supported mindfulness. It also does not explore the challenges that VR may introduce for individuals practicing mindfulness.

Although VR has the capacity to deliver support for mindfulness practice, it may also introduce its own unique challenges. For example, simulator sickness is a frequently reported negative side effect of VR, which produces symptoms similar to motion sickness [35,36]. Researchers have also speculated that headset discomfort may be a barrier to mindfulness in VR [24], but little is known about how specific elements of headset design (eg, comfort, weight, and degree of immersion) may impact the potential benefits of VR-supported mindfulness.

The existing evidence suggests that VR is a promising tool for supporting mindfulness. VR mindfulness interventions can lead to increased state mindfulness in both new and experienced meditator samples [12,22,24]. However, little is known about the end-user experience of using VR for mindfulness, in particular, user perceptions of *how* VR may aid or detract from a mindfulness practice. This is an important issue to explore when considering the range of potential mechanisms through which mindfulness may be supported in VR. A better understanding of mechanisms may inform design decisions (eg, environment types, the need for guidance, and target cohort) and subsequently impact the efficacy and potential use cases for such systems.

### This Study

This study aimed to explore how VR can support mindfulness practice and to understand user experience issues that may affect the acceptability and efficacy of VR-supported mindfulness. To achieve these aims, we conducted a pilot study in which 37 participants from the general population used a VR mindfulness app in a controlled laboratory setting. We sought to:

- Quantitatively assess changes in state mindfulness, emotion, and simulator sickness following the use of the VR app.
- Qualitatively explore the user experience of mindfulness in VR, with a specific investigation of whether particular features are helpful or disruptive to mindful awareness in this context.

## Methods

### Ethics Approval

The Swinburne University of Technology Human Research Ethics Committee (SHR 2018/256) and the University of Melbourne Human Research Ethics Committee (ID# 1852613.2) approved all the procedures.

### Description of the Virtual Reality Mindfulness App

This study used a VR mindfulness app that was designed for use in self-guided contexts. The app delivered a 15-min program of guided, focused-attention mindfulness within a VE created from an omnidirectional video footage of a forest. The VE included ambient audio (ie, sound originating from the forest) and a guided mindfulness voiceover. The app did not require the use of a hand controller, encouraging intuitive exploration between the user and the environment. The app was designed by the authors in collaboration with a commercial software company and it was developed in the Unity platform for use with the Oculus Go VR headset. During the design process, the app was refined through 3 focus groups (not reported here) in which 9 prospective users trialed the experience and gave feedback that helped address usability and content-related issues.

#### Omnidirectional Video and Audio

**Figure 1:**
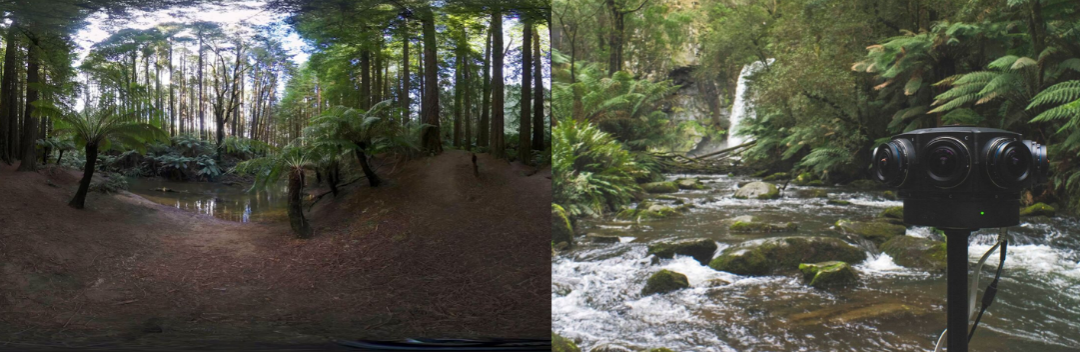
Filming locations for the virtual reality mindfulness app. The image on the left shows the environment presented for the first half of the mindfulness practice. The image on the right shows the second environment as well as the camera used to capture the omnidirectional footage.

The VE comprised forest scenes captured in the Great Otway National Park in Australia. The footage was recorded at two different sites in the forest, presented in Figure 1. The first site was a clearing near a river, and the second site was at the river’s edge. Both sites were filmed in 4K resolution using a Z Cam V1 Pro. The camera height was set at 1.3 meters, giving the impression that the user was experiencing the environment from a seated position. The footage did not include any visible people or animals, giving the impression that the user was alone in the environment. Ambient sounds of the natural environment were captured at the time of video recording, using omnidirectional and stereo microphones (Zoom H6 and Zoom H2n). This sound was overlayed onto the video footage during postprocessing.

#### Guided Mindfulness Voiceover

The VR app incorporated a guided mindfulness voiceover tailored to the VE that was delivered by a male narrator. The narrator is an experienced counseling and clinical psychologist with expertise in mindfulness-based interventions. The voiceover delivered a focused-attention mindfulness practice that used invitational language to guide the user’s attention to different parts of the VE (eg, “If you would like to...look at the rocks, you can explore their colour and texture”) and different physical sensations from the body (eg, “bring your attention to the breath”)*.* Guidance around thoughts and feelings was also provided (eg, “If you notice the mind has wandered...just gently bring your attention back to the moment”). The guided voiceover also allowed for periods of unguided practice (ie, without the voiceover), lasting up to 70 seconds.

### Participants

**Figure 2:**
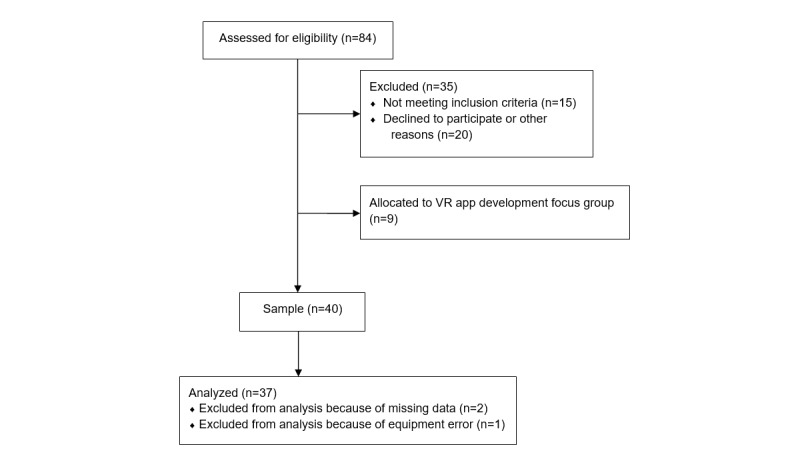
Flowchart illustrating participant recruitment to data analysis. VR: virtual reality.

Participants were recruited from the general population using a Facebook advertisement, a promotion on the website Mental Health Online, and flyers distributed around the Swinburne University campus in Melbourne, Australia. Potential participants completed a Web-based screening survey that assessed their eligibility for inclusion. The inclusion criteria required participants to be over the age of 18 years, have normal or corrected-to-normal hearing and vision, have no history of photosensitive epilepsy or previous experience of severe simulator sickness, and not be currently taking psychotropic medication or experiencing serious mental illness (eg, schizophrenia or psychosis). Figure 2 illustrates the recruitment process.

A sample of 40 participants trialed the VR app. As shown in Figure 2, a total of 3 participants were excluded from quantitative and qualitative analyses as they did not complete the full procedure (1 participant encountered a technical error with the VR app, and 2 participants did not complete posttest measures). Of the remaining 37 participants, 13 were men and 24 were women, with an average age of 37.86 years (SD 14.56; n=1 missing). A total of 4 participants had no previous experience of practicing mindfulness, but they had heard of it before. A total of 33 participants had previous experience of practicing mindfulness. Of these, 17 participants had tried mindfulness one to five times, and 16 participants reported regular mindfulness practice at the following frequencies: monthly (n=7), weekly (n=6), and daily (n=3). A total of 23 participants had never used VR or had only experienced it once, whereas 12 participants had tried VR several times and 2 participants reported using VR regularly, either less than monthly (n=1) or weekly (n=1).

A total of 19 of the 37 participants opted to contribute qualitative feedback at the time of recruitment. No significant differences, between participants who provided qualitative feedback and those who did not, were revealed in age (*U*=115.5; *P=*.15) or gender (χ^2^_1_=.1; *P*=.82). Owing to violations of chi-square test assumptions (expected cell count <5), further demographic comparisons were not performed. A visual inspection of the level of education, previous experience with mindfulness, or previous experience with VR suggested a similarity between groups, although the interviewees included all participants (n=4) without previous mindfulness experience.

### Materials

#### Measures

##### Demographics and Baseline Characteristics

Questionnaires were used to collect each participant’s gender, age, highest level of education, and previous experience with both VR and mindfulness—Response options: Not at all, I haven’t heard of it; Not at all, but I have heard of it; I have tried it once; I have tried it a few times (eg, two to five times); and I do mindfulness/VR regularly. Those who reported regular use of either VR or mindfulness were prompted to complete an additional item addressing frequency of use or practice—Response options: Less than monthly; Monthly; Weekly; Daily; and More than once a day).

Trait mindfulness was measured using a short-form of the Five Facet Mindfulness Questionnaire (FFMQ-15) [37,38]. The FFMQ-15 comprises 15 items drawn from the 39-item original measure, rated on a 5-point scale from “1=Never or very rarely true” to “5=Very often or always true.” A total score reflecting the tendency to be mindful ranges from 15 to 75. As a total scale, the FFMQ-15 demonstrated good reliability in this sample (Cronbach alpha=.83).

Participant baseline general mental health distress was assessed using the short-form Depression Anxiety and Stress Scale (DASS-21) [39]. In this sample, the DASS-21 showed good internal reliability (Cronbach alpha=.92).

##### Primary Outcomes

Change in state mindfulness was assessed using the 21-item State Mindfulness Scale [40], which was delivered before and after using the VR mindfulness app. Items are rated on a 5-point Likert scale from “0=Not at all” to “4=Very much” and address an individual’s level of perceived mindfulness in relation to the individual’s attention and meta-cognitive orientation across a recent period. The scale has a total score obtained by summing the item ratings (range 0-84). In this study, the State Mindfulness Scale had good internal reliability both before (Cronbach alpha=.95) and after (Cronbach alpha=.93) the use of the VR mindfulness app.

Emotion was measured dimensionally pre- and postapp use via 3 items—1 (Positive or pleasant), 2 (Negative or unpleasant), and 3 (Active or alert)—drawn from the circumplex model of emotion [41]. Participants were asked to consider how they were feeling “right now” and rated items on a 7-point Likert scale from “0=Not at all” to “6=Extremely.”

The Simulator Sickness Questionnaire (SSQ) [35] was used to detect changes in symptoms of simulator sickness. The SSQ comprises 16 items (eg, Eyestrain, Difficulty Focusing, and Fatigue), which are rated from “0=None” to “3=Severe,” and addresses the domains of nausea, oculomotor symptoms, and disorientation. It is important to note that there is considerable overlap between preexisting physiological and psychological symptoms (eg, anxiety symptoms) with the items on the SSQ [42]. For this reason, the symptoms of simulator sickness were measured pre and post use of the VR mindfulness app to determine a change in the symptoms relevant to the experience. In this sample, the internal reliability of the total score of the SSQ was poor both pre and post use of the VR mindfulness app (Cronbach alpha=.68 and Cronbach alpha=.51, respectively). As such, we considered the items as a checklist of symptoms rather than as a total scale, and we examined these individually as indicators of adverse responses.

##### Presence and General System Feedback

Presence was measured using a 22-item adapted version of the Presence Questionnaire 3.0 [29,43], which was shortened to reduce participant burden and remove items related to haptic senses and object manipulation, which were not features of the current system. As such, the total scale ranged between 22 and 154, with higher scores indicating a greater sense of presence in the VE. In this sample, the total scale demonstrated good internal reliability (Cronbach alpha=.84).

A total of 7 general system feedback items were also presented, relating to the perceived level of engagement, risk, quality, and future use (eg, “How engaging was the virtual reality mindfulness experience?”). These items were rated on a 5-point Likert scale, ranging from “0=Not at all” to “4=Extremely.”

### Procedure

All testing took place in a soundproof room at Swinburne University of Technology. Following informed consent, participants completed baseline questionnaires for approximately 15 min. Participants were then invited to wear an Oculus Go headset, which had the VR app preinstalled. Participants were seated in a swivel chair, giving them the ability to rotate their body to view the omnidirectional forest environment. A verbal check of visual clarity was conducted, and participants were assisted with adjusting the headset to improve clarity if required. The researcher then used the Oculus Go hand controller to start the VR mindfulness app (duration of approximately 15 min) and left the room. At the end of the experience, the participants removed the headset and the researcher reentered the room, at which time participants completed follow-up measures addressing state mindfulness, emotion, symptoms of simulator sickness, presence, and general feedback items (taking approximately 15 min).

For the 19 participants who opted to provide qualitative feedback, a semistructured interview, lasting an average of 26 min (range 16-46 min), was conducted. The interview questions focused on eliciting the participants’ views about the VR experience, positive and negative features of the app, and features of VR and VE, that helped or hindered the mindfulness practice.

All participants were thanked for their time and reimbursed with an Aus $10 (US $6.60) gift voucher (Aus $30 [US $19.79] if they had also provided qualitative feedback).

### Data Analysis

All quantitative data were processed and analyzed in IBM SPSS Statistics version 23.0. Descriptive statistics were used to describe the sample and calculate the ratings of general system feedback. Changes in mean scores from pre- to post-VR use on the State Mindfulness Scale [40] and emotion items were assessed using two-tailed repeated-measures *t* tests with an alpha level of .05. A Bonferroni-adjusted alpha level of .003 was applied to the analysis of the simulator sickness items on the SSQ [35]. Normality checks were conducted to confirm the appropriateness of a parametric model. Wilcoxon signed-rank tests were conducted where the assumptions were violated. Effect sizes were interpreted by following the guidelines from Cohen [44].

Qualitative data were collected by ES and 2 trained research assistants. The interviews were coded using a general inductive approach [45]. Both ES and RK conducted independent parallel coding by reading the transcripts separately and applying labels to the text through open coding. These codes were subsequently compared, discussed, and developed into a final set of 9 themes that characterized participants’ experiences [45].

## Results

### Quantitative Findings

Table 1 provides the mean scores and standard deviations of the primary outcome measures (state mindfulness, emotion, and arousal) both before and after using the VR mindfulness app. Trait mindfulness was normally distributed in the sample, ranging from 37 to 65 (mean 52.70, SD 7.50). On average, participants had low levels of general mental health distress, as measured by the DASS-21 (mean 9.19*,* SD 6.95; range 1-32).

**Table 1:** Descriptive statistics of primary outcomes pre- and post use of the virtual reality mindfulness app.

Variable	Pre-VR^a^ app use		Post-VR app use	
	Mean (SD)	Range	Mean (SD)	Range
State mindfulness	38.76 (18.04)	11-77	64.32 (13.34)	30-84
Positive emotion	4.32 (0.88)	2-6	4.78 (0.79)	3-6
Negative emotion	0.76 (0.90)	0-3	0.49 (0.61)	0-2
Arousal	4.00 (0.91)	2-5	4.19 (0.97)	2-6
Presence^b^	N/A^c^	N/A	114.83 (14.06)	81-149

^a^VR: virtual reality.

^b^Presence was measured after use of the virtual reality mindfulness app. This variable had n*=*1 missing observation because of an incomplete response.

^c^N/A: not applicable.

#### Primary Outcomes

A two-tailed repeated-measures *t* test was used to evaluate the change in state mindfulness following the use of the VR app. There was a statistically significant increase in state mindfulness scores from pre-VR to post-VR use (*t*_36_=–10.97; *P*<.001; 95% CI –30.30 to –20.84). The size of this effect was large, Cohen *d=*1.80.

Owing to non-normality, Wilcoxon signed-rank tests were conducted to examine the change in subjective positive and negative emotion as well as levels of arousal. There was a statistically significant increase in the median positive emotion rating from pre-VR (median*=*4.00; interquartile range [IQR] 4.00-5.00) to post-VR use (median*=*5.00; IQR 4.00-5.00; *Z*=–2.75; *P*=.006; *r*=.45). No significant changes were detected for negative emotion (pre-VR: median*=*1.00; IQR 0.00-1.00; post-VR: median*=*0.00; IQR 0.00-1.00; *Z*=–1.85; *P*=.06) or for arousal (pre-VR: median*=*4.00; IQR 3.00-5.00; post-VR: median*=*4.00; IQR 3.00-5.00; *Z*=–0.91; *P*=.36).

Table 1 also shows high average ratings of presence following the use of the VR mindfulness app, indicating that participants experienced a strong sense of *being there* in the forest environment.

#### Simulator Sickness

Mean scores of simulator sickness symptoms were low across all items, both before and after VR app use. All median scores were at or below the mild symptom awareness threshold (a score of 1 on the SSQ). Bonferroni-adjusted Wilcoxon signed-rank tests did not reveal significant differences in the SSQ items pre- to post-VR app use (all *P*>.003). At an individual level, no participant rated *severe* effects post-VR app use. Participants who reported symptoms in the *moderate* range post-VR app use included stomach awareness (n=2, change from *not at all* preintervention), fatigue (n=2, change from *not at all* and *mild* preintervention), general discomfort (n=1, change from *mild*), and difficulty concentrating (n=1, change from *mild*).

#### General Feedback Ratings of the Virtual Reality Mindfulness App

Overall, most participants reported that the VR mindfulness app was very engaging (22/37, 60%) or extremely engaging (10/37, 27%), that it had very intuitive interactions (19/37, 51%) or extremely intuitive interactions (13/37, 35%), and that it was very easy to use (16/37, 43%) or extremely easy to use (19/37, 51%). Most participants rated the quality of the visual and auditory experience as moderate (15/37, 41%) or very good (18/37, 49%). Participants also thought that the VR mindfulness app would benefit their mental health at a moderate level (11/37, 30%) to high level (14/37, 38%), with 19% (7/37) indicating that the VR mindfulness app would benefit their mental health extremely. Most participants indicated that they felt using the VR mindfulness app was not at all risky (35/37, 95%).

### Qualitative Findings

A total of 9 themes were produced from the participants’ qualitative feedback. These encapsulate descriptions of the general experience of mindfulness in VR, the main ways that VR helped or hindered mindfulness practice (presence and interruptions in presence, engagement, providing a scope for mind-wandering, directing and anchoring attention, and personalization), and perceptions of video fidelity and the headset.

#### Experiencing Mindfulness in Virtual Reality

Participants were positive about the mindfulness app, describing the experience it created as “comforting*,*” “calming*,*” “relaxing*,*” and “peaceful.” Expressions of positive emotion were common and often related to the feeling of being situated in a forest environment: “that specific stimulus is really comforting for me” [P20]. *Relaxing* was one of the most common descriptions: “I felt myself get really comfortable and kind of relaxed and engaged with the environment” [P30]. Participants commented on the tranquility of the VE: “I like sitting in the middle of a river or next to a creek bed or in the middle of the forest. It feels safe” [P7].

Another participant explained that a feeling of safety was related to being in an environment that “doesn’t change” and that “doesn’t feel like there’s all these people there” [P6].

Although most participants found the experience straightforward, some described taking time to adjust to VR. For some, more time was needed between putting on the headset and starting the practice:

there needed to be just a little more space between that direction and then...getting started because I was still fiddling around with the mask and trying to feel comfortable.

P9

For others, there was some apprehension around what they expected to see and do, although this period of apprehension did not appear to impact their practice:

[I]t was just that adjusting, but it wasn't really negative, it was just sort of a little bit of anxiousness. Am I doing it right? It took a few minutes to get used to it, but the scene...drew me in and I just forgot about it.

P2

#### Presence in the Forest Environment

Participants frequently mentioned experiencing a sense of presence in the virtual forest, which facilitated their ability to be aware of the present moment:

...to be able to be in a place that helps your mind focus in the present and in the now, something that's pleasant to look at, something that is sensory...it was actually helpful.

P9

it was just purely I was there and that was it.

P2

Some participants linked their sense of presence to feeling that their real-world concerns were reduced:

I think because you think you're there...it is hard to think about what's going on back in reality.

P27

Presence was often discussed in terms of being “transported” to the forest environment, which allowed participants to take a step back from their thoughts and daily life experiences. A participant described how:

it was great because it really kind of took me away from my thoughts...and just put me in this different world.

P30

Participants also noted that the *virtuality* of the environment helped facilitate this:

If I were sitting in that same environment in reality I would be thinking...are there other people there... is the car there. But knowing that this environment was virtual, I was able to simply enjoy it.

P9

#### Interrupted and Variable Presence

Although presence was an important precursor to engaging with mindfulness in VR, participants’ sense of presence appeared to vary dynamically while using the VR mindfulness app. A participant explicitly described how changes in their sense of presence acted as a distractor from the practice and sometimes caused brief disengagement from the experience:

parts of it felt like it was seamless. And then there's other parts where it felt like I could see that it was a projection. And then I was like oh that's really distracting because I'm looking at that as a projection.

P6

A participant described compensating for the loss of presence by actively redirecting attention within the VE, particularly to images that felt “more real” to them:

There was one occasion ... [where] there was something about the graininess or something about the image that made me go ‘oh yeah I'm not actually at the river’... I just would then focus my attention on something that felt more real...so it wasn't a big deal.

P42

The disparity between perceived presence and physical sensation was something participants described becoming aware of across the experience. Although this was not an issue for most participants, 1 individual claimed that this disparity was particularly disengaging:

they're not real sensations they're just the video of sensations...when you're in that place and you look down you don't see your body...that's unnatural...and it's a bit shocking and it kind of pulls you out of it a bit.

P39

The real-world environment also contributed to interruptions in presence and may have acted as a distractor to the mindfulness practice. This was primarily related to comfort within the physical space:

the chair was uncomfortable...sometimes the environment makes me forget about it. But then I was moving or the sound of the chair legs...it was kind of a break of my experience.

P18

if I was in like maybe the comfort of my own home...I'd be able to focus on my breathing.

P17

#### Engagement

Participants described the environment as being “engaging,” and this was discussed as a useful feature of the VE for practicing mindfulness:

I found the engagement in the images improved my mindfulness because I really did try to be present in the moment...it filled my mind.

P42

Presence was a prominent cause of being engaged:

because I thought ‘oh this is a real place’ I kind of was more involved in the little details of this place.

P30

A sense of being connected to the virtual experience also emerged from participants descriptions of being engaged:

I...felt really connected to it when I was watching really small details. ...the visual was really important for me.

P41

The experience of being engaged in the VE did not mean that it was “too grabby or like showbiz” [P24]. Rather, participants described the VE as having “not too much going on so it would distract you but enough to keep it interesting” [P3]. This appeared to support mindfulness practice by providing interesting stimuli in the environment, which helped to maintain attention in the present moment but without becoming boring or distracting:

I never felt like...I was getting restless or I needed to look at something else. I was quite happy where I was.

P34

#### Scope for Mind-Wandering

Part of the process when practicing mindfulness is the ability to become aware of mind-wandering and learning to direct attention back to the present moment [2]. Some participants described how their mind wandered in a conventional sense, involving thoughts related to their daily life or forthcoming events:

I went through a whole pile of other kind of recent events or things happening in my life.

P3

Other participants discussed how VR seemed to affect the content of their mind-wandering, such that this wandering became a response to stimuli in the VE:

[My mind] wandered within that world more than it wandered outside of it...because even though [the] mind wanders it still wanders within the parameters of what you're seeing.

P27

It's like wandering but wandering with focus...It was all very much focused but within the realms of the environment.

P30

#### Directing and Anchoring Attention

Similar to the use of an attentional anchor (eg, the breath) to maintain present-moment awareness in conventional mindfulness practices, participants described how they directed their attention to focus on “one thing” by anchoring to the elements of the VE: “Initially I'd have a look around and then I'd just focus on one particular bit I liked” [P42]. Different visual and auditory stimuli were described as helpful for anchoring to the present moment, with some participants describing how they focused solely on visual aspects of the VE to reduce distractions: “when I did find my mind-wandering...the visual side of it allowed me to...rein it in quicker” [P42]. Visual elements were cited as especially useful by participants who had previously struggled with mindfulness because of the difficulty of imagining a visual environment:

I've done meditation before and I just zone out to what they are saying...because your mind's working to picture something it then is working to daydream as well...Whereas, when it was just there in front of you, I think that it took a bit of pressure off of thinking, and you could be in the present.

P24

Two participants discussed how the omnidirectional video provided a prompt to “recenter” themselves:

My mind drifted less because I could just look at something then re-centre myself.

P42

I think being able to watch the water running when I was in that environment and then to be able to look up at the sky - that was really helpful in terms of a grounding experience.

P7

Variety in the visual environment was also perceived to be useful as it allowed participants to shift their focus:

You just notice different things because you have that space to kind of focus on that, or the ability to kind of go oh I'm bored of that rock, I'll go check out the tree over there.

P26

Other participants described how the environment’s sounds were more useful to them. For some, this occurred within a process of becoming aware of the full environment:

I love to hear those sounds so sometimes just bringing my attention back to the sounds, which then brought my attention back to the visuals and then I was in that entire environment again.

P9

Some even chose to close their eyes to focus on the sounds:

I actually shut my eyes a lot...I felt like I'd focused on myself and then focused on the environment and then I like to focus on the sounds because they kept going and that was really good for me.

P6

Regarding the guided mindfulness voiceover, 15 participants found this to be a useful feature of the VR app. They described how the narrator’s suggestions “provided ways to sort of dig deeper into the experience” [P27] and how they were able to return to the present moment during mind-wandering so they would not “drift into that space where you're just observing it just in awe” [P2]. However, 5 participants who found the voiceover useful also commented that it became a source of distraction. A total of 2 others described the voiceover as entirely distracting. One issue was that the frequency of suggestions did not always allow for the time to explore and attend to the VE:

it'd be like “Okay now look at this, now look at this”...I [had] just started looking at one thing, now I can't not focus on the other thing.

P3

Another was that some people did not need the voiceover because they had previous experience with mindfulness: “I do my own silent practice...the audio guide of meditation now I find distracting” [P34].

Interestingly, participants sometimes described becoming aware of what was *not* in the VE (eg, a sense of touch and smell congruent with a forest) and how the absence of information directed their attention during the practice. A participant used audio as a substitute for touch:

I wish I could touch the water just to feel if it's cold ... I guess that was the reason that I focused more on the audio.

P18

Another participant described missing sensations and how this was interwoven with the process of guiding attention back to the present moment:

there were moments where I was able to bring myself back to the actual moment...and I had this sense that I really wanted to...actually feel the breeze. I was missing that sensation...I actually was very aware of the fact that sensation...was missing...

P9

Participants also commented on the way in which features of the VR app combined to guide attention back to the present moment. By providing many sources of detail to which to attend, the app’s “multi-modal” nature was seen as a benefit, and participants discussed the complex and evolving integration of multiple sources of stimuli:

It brings the ambience of the environment and the mindfulness into your actual body as opposed to just your mind, so you're focusing not only on the VR experience but also what your body is doing and then how it relates to the VR experience as well, so it's all connected.

P26

This process of becoming aware of and incorporating the experience of multiple senses was also described in relation to understanding and shifting between the different sources of information presented by VR and felt by the body:

You can feel your feet on the ground, you can feel the back of the chair, you know you're not in this environment. And because you're being present in the moment you've kind of got this contrast of sensations...On the other hand you open your eyes and...you feel part of the environment again...perhaps when you close your eyes...you become more aware of the chair again....

P20

#### Personalization

Participants’ responses on the use of attentional anchors highlighted the potential of VR to support users in personalizing their practice. Participants frequently described how they chose to attend to parts of the experience that they enjoyed. In this way, personal preferences for visual and auditory stimuli may have been directing attention and maintaining engagement with mindfulness practice:

I could acknowledge and recognise in myself the parts of the environment that I really enjoyed and I sort of stuck to looking at them and looking at all the detail in them.

P27

The freedom to focus on particular elements of the VE also appeared to support a sense of agency:

...I'd find moments where I was staring up at the sky...counting my breaths...It felt like it was an active choice...engaging with this in your own time and of your own volition.

P7

Agency was considered a positive aspect of the VR mindfulness app, and it incorporated discussion of emotion:

If there's an area I don't really feel like it's calming me or I don't really feel a connection to...I just like twirl around and move somewhere else. So I just felt like I was able to control my own experience...I could...control what I was feeling.

P17

However, participants who reported practicing mindfulness regularly stated that the features of VR sometimes competed for their attention:

I found it quite distracting from what I was used to. Because it was an additional sensory distraction rather than trying to find a single point to focus. So it was a little confusing for a lot of it.

P34

This preference for a *single point to focus* was reinforced by another participant who valued having a “simple focus of attention...something to crystallise my attention around...[to] forget about the periphery” [P20]. These comments may suggest the need for variable stimulus levels or options within a VE, particularly when considering a user’s previous mindfulness experience.

#### Perceptions of the Video Fidelity

Visual quality was an important feature to participants, which impacted their ability to focus on the mindfulness practice. Although our app used professional-quality video, 2 participants noted discomfort created by bright or blurry images:

like when you look into the sun and how it burns your eyes it's kind of like that kind of type of effect.

P3

The one thing that I found that kind of like impeded the experience was that when you try to look closely at something, the resolution of the display doesn't really allow you.

P40

A participant described how sections of the environment, which appeared to be of lower quality (eg, as they were further away or blurry), were distracting and how they actively redirected attention to parts of the environment with higher fidelity:

I kind of just stopped paying as much attention [to] things which were distracting...I feel like [things that] were kind of higher quality or less far away...wasn't as much of an issue.

P3

#### Perceptions of the Headset

In reflecting on the experience, participants indicated that the distractions introduced by the headset were both infrequent and minor. A majority of participants reported that it was “not really uncomfortable” [P34] or that “You kind of stop paying attention to it” [P3] when engaging with the VE. This may be because our app was delivered using a commodity headset (Oculus Go) that has been designed for user comfort.

The participants who did mention distraction from the headset noted issues of physical discomfort and disruption to immersion. Regarding physical discomfort, 5 participants described the headset as feeling heavy or tight on the head. A total of 2 participants described becoming aware of this sensation during the mindfulness practice. The first participant described how the awareness of the headset introduced a challenge for engaging with the VE:

it wasn't a case of trying to pull myself back to being mindful because of my thoughts, it was pulling myself back...into the virtual world away from the thought of oh this thing is heavy on my face.

P9

The second participant mentioned how the tension between being in the VE and “attempting to ignore the headset...puts a bit of a load on your mental state” [P40]. Other participants seemed to actively incorporate their awareness of the headset as a focus for their attention: “I was sort of focusing on [the headset] sometimes instead of my breathing...” [P14].

Regarding disruption to immersion, participants commented on the way the headset sat on the head and how this allowed light from the external environment to seep in to the bridge of the nose. This was described as a disengaging experience, making it “really hard...to concentrate” [P18] for one participant. For another, the ability to see his or her (real) legs when looking down was “a bit off-putting...[and] distracting” [P32]. They discussed a set of pragmatic actions to reduce the impact of the headset, which included “ma[king] a conscious effort to look left, look right, look up, and look straight ahead but not really right down” [P32].

## Discussion

### Principal Findings

This study aimed to explore how VR can support mindfulness practice and to understand user experience issues that may affect the acceptability and efficacy of VR mindfulness in the general population. To achieve these aims, we conducted a pilot study in which 37 participants used a VR app in a controlled laboratory setting.

Our first main finding was a large and statistically significant increase in state mindfulness, indicating that the VR app we created was successful in supporting mindful awareness in our sample. This finding is consistent with previous studies [12,24], and this provides further empirical support for the ability of an appropriately designed VR app to enable mindfulness practice. Qualitative feedback from participants suggested that the features of the VR app, including the VE and guided voiceover, played an active role in bringing their attention to the present moment. Although this points to the utility of VR for supporting mindful awareness, it is important to note that VR provides users the opportunity to engage in two “present moments”—one that is simulated and the other in the real world. Our participants commented that present-moment awareness often shifted between the two worlds, in line with guidance from the mindfulness audio. For some, this was experienced as a distraction, and for others, these dual present-moment realities were actively integrated. This may help to create a mindful state by shifting attention to the present-moment experience both within and outside of the VE, and future research involving comparisons with other groups can help to further unpack the unique role of VR in contributing to changes in state mindfulness [24].

Our second main finding was a statistically significant increase in positive emotion following the use of the VR mindfulness app. No change was indicated for negative emotion, likely because of floor effects. Similarly, there was no change in arousal. However, the participants qualitatively described a sense of “relaxation.” Although mindfulness practice does not seek to engender a positive mood state, the ability of a VR app to facilitate positive emotion may usefully contribute to mindfulness practice by reducing experiential challenges related to negative emotion [46]. This may be an important consideration for novice meditators or for groups of meditators who experience complex or negative emotions [20], and this may further enhance the acceptability of VR for mindfulness practice. However, it is doubtful that positive affect arises simply from the use of VR, and therefore, the content in the VE presented to the user is likely to play an important role. In our VR app, positive affect may have arisen from the simulated forest environment, which participants found to be peaceful and enjoyable. Natural spaces have been shown to provide individuals with well-being benefits [47], and this effect has been observed in the use of VR and other nonimmersive platforms (ie, computer screens) [48,49]. Future work should explore the impact of other kinds of neutral and positive VEs to explore the role of positive affect in supporting mindfulness practice in VR.

Our third main finding was that the VR mindfulness app did not generate any severe adverse experiences. Simulator sickness has been noted as a potential risk factor for VR in general [36], but our quantitative and qualitative data indicated only minor symptom increases for some participants. These increases were related to discomfort, concentration, and fatigue, and these may have been influenced by the image quality (particularly blurriness) or by the intensity of light in some parts of the VE. However, these are modifiable aspects of the experience; thus, these can be alleviated with the careful refinement of a VR app. In addition, we found that only limited discomfort was introduced by the Oculus Go. This indicates that the weight imposed by a commodity VR headset does not act as a substantial barrier to engagement, provided that this weight is minor. These practical insights are important as they emphasize the potential for a well-designed VR app to be a safe, acceptable, and tolerable approach for supporting mindfulness practice; they are also important for highlighting the areas for consideration (ie, image quality and brightness) in risk minimization. It may be especially important to consider these issues when designing VR apps for use with clinical populations.

### How Virtual Reality Can Support Mindfulness Practice: Presence, Attention, and Agency

Our qualitative findings provide insight into the mechanisms that may have contributed to an increase in state mindfulness among our sample and into the potential affordances of VR to mindfulness practice. The key features mentioned by participants were the sense of being present in a calm environment that provided a bounded scope for attention, availability of structured guidance along with the freedom to explore, and ability to utilize multiple attentional anchors within the VE and in line with personal preferences.

The sense of presence emerged as a perceived support to mindfulness practice in VR, and its disruption became a temporary distraction. Our participants discussed how being in the VE allowed them to step back from everyday concerns and created a bounded scope around the content of their mind-wandering. Being engaged in the VE was beneficial in that it maintained interest across the practice, and the VE itself was neither overwhelming nor burdensome on concentration for most participants—a challenge often reported with conventional mindfulness practice [10]. Participants described a process of constructing the realness or believability of their experience by attending to parts of the VE that were appealing to them. Even when the sense of presence was interrupted (eg, by real-world intrusions), participants described directing their attention back to the VE and choosing to become involved in the simulation again. It could be that constructing a sense of presence, a process intrinsic to immersive VR, is a useful feature for training present-moment attention by actively involving attention shifting and facilitating engagement in the moment.

The availability of the multiple types of stimuli to act as anchors for present-moment attention was also identified as beneficial by our participants. In addition to the contribution that anchors made to support attention in the present moment, they provided users with an opportunity to personalize their experience by directing attention to their preferred objects of interest, which may have consequently contributed to a feeling of agency. As discussed by Anderson and Farb [3], individuals hold personal preferences for attentional anchors. Catering to preferences or motivations to engage with an attentional anchor may thus improve engagement with and adherence to mindfulness practice [3]. Peters et al [50] argue that designing (digital) interventions with the intention of satisfying basic psychological needs (self-determination: autonomy, competence, and relatedness) [51] at the point of use (ie, the interface) may greatly improve motivation, engagement, and general well-being. Although conventional mindfulness practices may support feelings of agency as an outcome of practice over time and as experience increases, the freedom of movement of attention within VR may uniquely contribute to a sense of agency within a practice (ie, at the point of use). This feature may motivate sustained use [50].

Kaplan’s [52] Attention Restoration Theory (ART) provides a useful framework for understanding how the features of VR, which were discussed by our participants, may be involved in facilitating present-moment awareness and contributing to the positive emotion. ART is a complementary viewpoint to mindfulness, which suggests that directed attention fatigue impacts mental health (eg, stress) and that person-environment interactions contribute to the recovery of attention resources, which may have mental health benefits. Environments that are considered restorative in ART are hypothesized to contain properties that involve (1) “being away” from an individual’s everyday environment (in our data—transportation), (2) containing features that hold attention with little effort via fascination (eg, nature), (3) containing features that maintain engagement and have coherence and scope (in our data—multiple types of stimuli to act as attention anchors), and (4) containing elements that support “what one wants or is inclined to do” (in our data—personalization) [52]. These properties were reflected in participant comments, and taken together, these suggest that the person-VE interaction may support a restorative experience and augment the mindfulness practice.

### Considerations for Supporting Mindfulness in Virtual Reality

From the discussion of the mechanisms that supported mindfulness practice in VR, several design considerations emerge, which can inform future VR-supported mindfulness practices. First, an appropriately designed environment that supports the active construction of a sense of presence may be beneficial, both in implicitly guiding attention to the VE and in supporting the properties of a restorative environment. Consistent with ART [52], participants described the environment as having an appropriate degree of engagingness (not too interesting but interesting enough), which aided in their ability to maintain present-moment awareness. Where this consideration may need to be modified, however, is for more experienced meditators, who perceived the VE to be confusing or overstimulating due to the variety of anchors available. Second, the capacity of a VE to support personalization through providing a choice of attentional anchors both supports engagement and mitigates the need for extensive customization of a VR experience beyond an appropriate choice of VE. In the VE, users are likely to gravitate to the elements that best support mindful awareness and their sense of presence.

Design considerations also emerged from our participants’ description of distracting and disengaging experiences. Demands introduced by the physical environment have been discussed in previous research as a prominent challenge in conventional meditation practice [10] (although this should be balanced with an understanding that a part of mindfulness practice is concerned with noticing when the mind is distracted and bringing attention back to the present). In our app, distractions in the physical environment were not necessarily removed by the use of VR, as participants discussed experiences of feeling disengaged when they became aware of real-world stimuli. Despite this, these experiences were not so distracting as to completely disrupt the mindfulness practice, indicating that the system could help reduce potentially disruptive environmental distractors if it were used outside the lab. We also noted that distractions were experienced in the VE (eg, the environment being overwhelming and footage quality requiring greater concentration). This suggests the potential to consider VE design from the perspective of generating environments that are variably distracting in a graded approach for training mindful awareness.

### Strengths and Limitations of This Study

A strength of this study was the mixed methods approach to examining the experience of a VR mindfulness app. This approach provided the opportunity to compare the quantitative measures of efficacy with the findings of previous studies utilizing different VR designs [12,24], while also qualitatively exploring the practice of mindfulness in VR and asking, from a user’s perspective, what helps and what hinders.

Our measures of efficacy were limited to state-based change in mindfulness and emotion following a single session of VR use in a controlled lab setting. As such, we cannot generalize the changes in mindfulness and emotion to repeated use contexts (eg, in the real world) or to long-term outcomes relevant to mental health (eg, changes in trait mindfulness and emotion regulation). More research is required to understand the patterns of engagement and mental health outcomes that arise from ongoing use of a VR mindfulness app. Our work provides the grounding for these lines of study.

Furthermore, without a comparison or control group, it is unclear whether the changes we observed in state mindfulness and emotion are primarily driven by contributions from the VR mindfulness app. Present-moment awareness may have increased in response to phenomena outside of the app. Although we argue that VR was integral to shaping participants’ experience, comparing virtual and real-world mindfulness practice may help to further understand the unique contributions a VR system can have for the user. Finally, our capacity to examine a range of moderators (eg, level of previous experience with mindfulness) was limited by sample size. Future work may want to consider how individual characteristics impact the responses to VR mindfulness practice in an effort to identify the most beneficial use cases.

### Conclusions

Mindfulness practice can benefit mental health by enabling people to adopt an open, accepting, and nonjudgmental attitude toward present-moment experience. Through the use of a VR app, this study explored how VR can support mindfulness practice, and it investigated user experience issues that may help or hinder the ability to practice mindfulness in VR.

We found that VR can support statistically significant increases in state mindfulness and positive emotion in participants from the general population without fostering negative emotions or heightening arousal. The VR app we studied was well received and did not induce any strong symptoms of simulator sickness. These findings suggest that VR can foster mindfulness in a way that is safe and enjoyable, evidencing the acceptability and efficacy of VR as a platform for mindfulness.

We also found that the sense of presence in the VE and the freedom to explore were key contributors to participants’ experience of mindfulness. The VE allowed participants to select anchors of their choice which enabled them to align with or freely deviate from the structured audio guidance provided in the app. The app also played a role in restricting the scope of participants’ mind-wandering, enabling them to gently return to the present moment within the virtual forest setting. These features speak to the potential of VR to minimize the potential affective and task demands that are often experienced as challenges to mindfulness, by allowing users to personalize their practice in response to the content of the VE. However, our study revealed that video fidelity, level of guidance, and breaks in presence were sometimes perceived to be disengaging. These issues should be accounted for in future apps that are designed to support VR mindfulness.

Our investigation suggests that VR is able to combine a guided mindfulness practice with exposure to a relaxing, restorative environment. These complementary features may be a unique affordance of a well-designed VR app. Further research is required to compare the utility of this approach with conventional mindfulness practice.

## Abbreviations

ART: Attention Restoration Theory
FFMQ-15: Five Facet Mindfulness Questionnaire
SSQ: Simulator Sickness Questionnaire
VE: virtual environment
VR: virtual reality
